# Usefulness of severity criteria in DSM Eating Disorders and existing gaps

**DOI:** 10.1192/j.eurpsy.2025.170

**Published:** 2025-08-26

**Authors:** F. Fernandez-Aranda, S. Jimenez-Murcia

**Affiliations:** 1 Clinical Psychology, University Hospital of Bellvitge, Barcelona, Spain

## Abstract

**Abstract:**

Anorexia nervosa (AN), bulimia nervosa (BN) and Binge Eating Disorders (BED) are diagnosed using the DSM-5 (American Psychiatric Association [APA] 2013) and its revised text (DSM-5-TR; APA 2022) or the ICD-11 (World Health Organization [WHO] 2019). While both systems use a severity indicator for AN based on body mass index (BMI), only the DSM-5 proposes a severity indicator for BN, based on the weekly frequency of purging behaviours, and, in the case of BED, based on the weekly frequency of binge episodes. These severity scores aim to index the level of general and eating disorder (ED) psychopathology, and thus, if valid, should serve as a crucial tool for tailoring treatment decisions, including duration, frequency and type of treatment. However, the current literature is contradictory with regard to the appropriateness of the criteria used and their validity. Therefore, alternative criteria have been proposed (e.g. drive for thinness, duration of the disorder…), especially when exploring chronicity and long-lasting EDs. Furthermore, few studies have directly examined the relationship between severity ratings and treatment outcome. In this presentation, we will discuss current gaps in severity indicators and potential alternatives. Data from existing longitudinal cohorts will be described.

**Disclosure of Interest:**

F. Fernandez-Aranda Consultant of: Novo Nordisk. It has no conflict of interest in the current talk. Nothing about pharmacological prescription will be discussed in my talk., S. Jimenez-Murcia Consultant of: Novo Nordisk. It has no conflict of interest in the current talk. Nothing about pharmacological prescription will be discussed in my talk.

